# Beyond Inflammatory Ratios: Age and CRP as Key Predictors of Re-Amputation in Diabetic Foot Patients

**DOI:** 10.3390/metabo16100778

**Published:** 2026-10-14

**Authors:** Huseyin Bahadir Gokcen, Alp Yekta Gokalp, Sezgin Bahadir Tekin, Gizem Tekin, Onur Mercan, Umut Kahilogullari

**Affiliations:** 1 Department of Orthopedics, Medicana International Hospital, 34520 Istanbul, Türkiye; bahadrgokcen@gmail.com; 2 Department of Orthopedics, Nigde Omer Halisdemir University Research and Practice Hospital, 51100 Nigde, Türkiye; 3 Department of Orthopedics, Medical Point Hospital, 27584 Gaziantep, Türkiye; sezginbahadirtekin@gmail.com; 4 Department of Internal Medicine, Gaziantep 25 Aralık State Hospital, 27090 Gaziantep, Türkiye; gizemhacimustafaoglu@gmail.com; 5 Department of Orthopedics, Birecik State Hospital, 63400 Sanliurfa, Türkiye; onurmercan.md@gmail.com; 6 Department of Orthopedics, Gaziantep University Hospital, 27310 Gaziantep, Türkiye; kahiloglu327@gmail.com

**Keywords:** diabetes complications, diabetic foot, amputation, revision surgery, serum markers

## Abstract

**Background and Objectives:** Re-amputation is an important adverse outcome of diabetic foot disease. This study compared age and preoperative inflammatory parameters between patients with and without subsequent re-amputation and examined their adjusted associations with re-amputation group membership. **Materials and Methods:** The records of 206 patients treated between 2013 and 2024 were reviewed retrospectively, with 103 patients included in each group. Laboratory parameters obtained before the index amputation were analyzed in both groups. Between-group comparisons were performed using the Mann–Whitney U test. Three exploratory binary logistic regression models included age and C-reactive protein (CRP), together with the neutrophil-to-lymphocyte ratio (NLR), monocyte-to-lymphocyte ratio (MLR), or platelet-to-lymphocyte ratio (PLR). The regression and ROC analyses were based on 197 complete cases. **Results:** Patients in the re-amputation group were older and had higher WBC, neutrophil, monocyte, CRP, NLR, MLR, and PLR values and lower lymphocyte values. In the primary model, age (adjusted OR = 1.034, 95% CI: 1.010–1.058; *p* = 0.006) and CRP (adjusted OR = 1.006 per 1 mg/L, 95% CI: 1.003–1.010; *p* = 0.001) were independently associated with re-amputation group membership, whereas NLR was not (*p* = 0.834). Neither MLR (*p* = 0.176) nor PLR (*p* = 0.446) was independently associated in the alternative models. Individual-marker AUCs ranged from 0.604 to 0.647, indicating limited discrimination. **Conclusions:** Older age and higher pre-index-amputation CRP levels were independently associated with membership in the re-amputation group. Although NLR, MLR, and PLR differed between groups, these ratios were not independently associated after adjustment for age and CRP. The limited discriminatory performance and data-derived cutoff values require validation in larger, independent cohorts.

## 1. Introduction

Diabetic foot is recognized as the primary cause of non-traumatic amputations, with approximately 75% of lower extremity amputations performed due to this condition [1]. Individuals with diabetes have a 25% probability of developing foot ulcers related to their condition during their lifetime [2]. The development of diabetic foot ulcers is thought to be caused by a combination of peripheral vascular disease, peripheral neuropathy, and infection [3]. Although diabetes and peripheral arterial disease (PAD) frequently coexist, the primary indication for amputation should be clearly distinguished between diabetic foot-related complications and arterial insufficiency attributable to an isolated arterial occlusion. In diabetic foot disease, peripheral neuropathy increases the likelihood of unrecognized trauma and repetitive pressure, while impaired perfusion limits the oxygen and nutrients needed for tissue repair. Superimposed infection can further exacerbate tissue damage and prolong inflammation, thereby impairing wound healing [4]. In addition, advanced peripheral arterial disease may compromise tissue perfusion, leading to ischemic rest pain that is often more pronounced at night. By contrast, loss of protective sensation in neuropathic diabetic foot ulcers may allow tissue injury to progress with little or no pain, delaying its recognition. However, neuropathy and arterial disease frequently coexist in diabetic foot disease, and neuropathy may mask ischemic pain even when perfusion is severely impaired. Therefore, the absence of pain should not be considered a reliable clinical criterion for differentiating diabetic foot complications from arterial insufficiency [4]. Additionally, patients who have undergone amputation due to diabetic foot may necessitate re-amputation because of the impaired healing process. The re-amputation rates for patients who have had surgery for diabetic foot were reported to be 26.7% at one year, 48.3% at three years, and 60.7% at five years [5]. Although re-amputation poses a significant issue for patients with diabetic foot ulcers, we currently do not possess a biomarker that can predict this outcome.

White blood cell (WBC), lymphocyte (LYM), neutrophil (NEU), monocyte (MON), and platelet (PLT) counts are readily obtained from a routine complete blood count, while NLR, MLR, and PLR are calculated from these counts. Together with separately measured C-reactive protein (CRP), these parameters have increasingly been investigated as markers of systemic inflammation. Selected inflammatory markers have also been studied in relation to diabetic neuropathy [6] and diabetic retinopathy [7]. Previous studies also have associated these inflammatory markers with the risk of diabetic foot ulceration (DFU), lesion severity, amputation, short- and medium-term mortality [8]. However, it remains unclear whether blood-cell ratios measured before the index amputation are associated with subsequently documented re-amputation after accounting for age and CRP. This study therefore compared age and preoperative inflammatory measures between patients with and without documented re-amputation and explored the associations of NLR, MLR, and PLR in separate models that included age and CRP.

## 2. Material and Methods

In this retrospective study, the medical records of patients who underwent amputation or re-amputation surgery for diabetic foot at Gaziantep University Hospital between 2013 and 2024 were reviewed. Patients who met the predefined eligibility criteria were selected from the hospital database, and 103 patients were included in each group, resulting in a final study population of 206 patients. The first group consisted of patients undergoing primary amputation, whereas the second group included patients undergoing re-amputation. Patients with insufficient data to assess eligibility or determine outcome classification were excluded from the study. Missing data for individual laboratory parameters did not lead to exclusion from the overall study cohort; analyses involving these parameters were performed using the available data. No matching procedure was performed between the study groups. The study was approved by the Gaziantep University Non-Interventional Clinical Research Ethics Committee (Decision No. 2025/188) and was conducted in accordance with the Declaration of Helsinki. Eligible patients were aged 18 years or older and had undergone an index lower-extremity amputation because of diabetic foot complications. Patients who subsequently underwent a more proximal amputation on the same limb were classified in the re-amputation group. Within each eligible outcome group, patients were assigned computer-generated random numbers using the RAND function in Microsoft Excel (version 2021, Microsoft Corp., Redmond, WA, USA), sorted according to these numbers, and the first 103 patients in each group were selected. This balanced sampling strategy was designed to facilitate between-group comparisons and does not estimate the population frequency of re-amputation. Patients were excluded if the amputation was attributable to trauma, congenital deformities, tumors, or other malignancies. Patients whose primary indication for amputation was an isolated arterial occlusion documented by angiography or Doppler ultrasonography, rather than diabetic foot-related complications, were excluded. Patients with Buerger’s disease (thromboangiitis obliterans) or other systemic vasculitides were also excluded, as were those with a history of extremity surgery unrelated to amputation.

Patients were categorized according to re-amputation status documented in the available hospital records after the index amputation. Group 1 comprised patients with no documented subsequent amputation at a more proximal level on the same limb, whereas Group 2 comprised patients with such a procedure. Amputation was defined as the surgical removal of any portion of a limb. Contralateral amputations, repeated procedures at the same anatomical level, wound debridement, secondary suturing, and stump revisions without progression to a more proximal amputation level were not classified as re-amputations. In both groups, laboratory parameters were obtained from blood samples collected within 24 h before the index amputation; thus, all measurements preceded any documented re-amputation.

Re-amputation status was ascertained from the hospital records available for review. A uniform follow-up period was not established, and time from index amputation to the last available record was not consistently available for analysis. Accordingly, Group 1 indicates no documented re-amputation in the available records, rather than confirmation that re-amputation never occurred. Time-to-event analyses were not performed.

Patient age and preoperative laboratory parameters, including WBC, CRP, LYM, NEU, MON, PLT, NLR, MLR, and PLR, were recorded and compared between the study groups. NLR, MLR, and PLR were calculated from the absolute blood-cell counts as NEU/LYM, MON/LYM, and PLT/LYM, respectively.

Owing to the retrospective design, some laboratory measurements were not available for all patients. CRP values were missing for nine patients, all of whom were in the group without re-amputation. These patients were retained in the overall study cohort and included in analyses for which CRP data were not required. No imputation of missing data was performed. For the multivariable logistic regression analysis, a complete-case approach was used; patients with missing data for any variable included in the model were excluded from that specific analysis only.

### Statistical Analysis

Before patient sampling, an a priori sample-size calculation was performed using G*Power version 3.1.9.7. The calculation was based on the difference in CRP levels between patients with and without re-amputation reported by Gülcü et al. [9]. In that study, the reported CRP values were 4.2828 ± 3.53533 mg/dL in patients without re-amputation and 6.1090 ± 4.24402 mg/dL in patients with re-amputation. These values yielded a pooled standard deviation of 3.6968 mg/dL and a standardized mean difference in Cohen’s d of 0.494. Using a two-tailed independent-samples t-test, an α error probability of 0.05, a statistical power of 90%, and a 1:1 allocation ratio, the minimum required sample size was calculated as 176 patients, with 88 patients required in each group. After the final sample had been established, a post hoc power calculation was performed using the same effect size, test specification, and α error probability, with 103 patients in each group. The resulting power was 94.2%. This retrospective power estimate applies exclusively to the between-group comparison used for sample-size planning and was not used to assess the adequacy, stability, or performance of the multivariable logistic regression models.

Continuous variables were summarized using mean ± standard deviation and median with the 25th and 75th percentiles. Between-group comparisons were performed using the Mann–Whitney U test and all observations available for each variable. To examine the observed pattern of missing CRP data, age, WBC, NLR, MLR, and PLR were compared between patients with missing (*n* = 9) and available CRP measurements (*n* = 94) within the group without re-amputation.

No missing values were imputed in the primary analyses. As a sensitivity assessment, the primary logistic regression model was refitted in two scenarios in which the nine missing CRP values were assigned either 12.75 or 266.60 mg/L, the approximate values of the 10th and 90th percentiles of observed CRP values in the group without re-amputation. Observed CRP values were unchanged, and each scenario model included all 206 patients.

Variables demonstrating significant between-group differences in the univariate analyses were considered potential candidates for multivariable modeling. Because several absolute leukocyte counts and inflammatory ratios were mathematically and biologically related, their simultaneous inclusion could have introduced redundancy and collinearity. Age, CRP, and NLR were therefore selected for the primary exploratory model based on the observed group differences, clinical relevance, and previous literature [10,11]. NLR was chosen as the representative cell-count ratio because it has previously been investigated in diabetic foot cohorts; however, this exploratory selection did not imply superiority over MLR or PLR. Two additional exploratory sensitivity models were consequently fitted by replacing NLR separately with MLR and PLR while retaining age and CRP. Absolute leukocyte components and multiple inflammatory ratios derived from overlapping blood-cell counts were not entered simultaneously, and no automated variable-selection procedure was used.

All three binary logistic regression models were fitted using the Enter method, with all specified predictors entered simultaneously and retained throughout the analysis. Re-amputation status was coded as 0 for no re-amputation and 1 for re-amputation. Age was modeled per one-year increase, CRP per 1 mg/L increase, and each inflammatory ratio per one-unit increase. Because CRP was included in all three models, complete-case analysis was applied. The resulting analysis sample comprised 197 patients, including 103 patients with re-amputation and 94 patients without re-amputation.

Overall model significance was evaluated using the omnibus likelihood-ratio chi-square test, and Nagelkerke R^2^ was reported as a measure of explained variation. The Hosmer–Lemeshow goodness-of-fit test was used to assess evidence of lack of fit. Multicollinearity was evaluated using tolerance and variance inflation factor values obtained from auxiliary linear regression analyses performed using the same complete-case sample. Tolerance values greater than 0.20 and VIF values below 5 were considered indicative of the absence of problematic multicollinearity.

Linearity of the continuous predictors in the logit was evaluated using Box–Tidwell interaction terms. Because three continuous predictors were evaluated within each model, a Bonferroni-adjusted significance threshold of 0.0167 was applied to the interaction terms. Standardized residuals, Cook’s distances, and leverage values were examined to identify potentially influential observations. Standardized residuals with an absolute value greater than 3 and Cook’s distances greater than 1 were considered potentially influential. A leverage screening threshold of 2(k + 1)/*n* = 0.0406 was used for the primary model, where k represented the number of predictors and *n* represented the analysis sample size. A sensitivity analysis excluding the observations with a Cook’s distance greater than 1 was performed to evaluate the stability of the primary model findings.

ROC curve analysis was performed using the same complete-case sample of 197 patients for age, CRP, NLR, MLR, and PLR, with re-amputation defined as the positive state. Areas under the curve were estimated using the nonparametric method and are presented with standard errors and 95% confidence intervals. Optimal cutoff values were selected by maximizing the Youden index, and the corresponding sensitivity and specificity values were calculated. Because these cutoff values were derived and evaluated in the same sample, they were considered exploratory.

All statistical analyses were conducted using SPSS version 26.0 (IBM Corp., Armonk, NY, USA). Unless otherwise specified for the Box–Tidwell analyses, all tests were two-sided, and *p* < 0.05 was considered statistically significant.

## 3. Results

### 3.1. Clinical and Laboratory Characteristics Associated with Re-Amputation

#### 3.1.1. Descriptive Statistics and Intergroup Comparisons

We first assessed whether missing CRP data showed an apparent systematic pattern within the group without re-amputation. Patients with missing CRP values (*n* = 9) did not differ significantly from those with available CRP values (*n* = 94) with respect to age, WBC, NLR, MLR, or PLR (all *p* = 0.414–0.788). In the main between-group analysis, patients who underwent re-amputation were significantly older than those who did not (70.08 ± 13.07 vs. 65.18 ± 12.91 years, respectively; *p* = 0.009). They also had higher WBC (*p* = 0.006), neutrophil (*p* = 0.002), monocyte (*p* = 0.040), and CRP levels (*p* < 0.001), whereas lymphocyte levels were lower (*p* = 0.031). NLR (*p* < 0.001), MLR (*p* = 0.004), and PLR (*p* = 0.005) were also higher in the re-amputation group. Platelet counts did not differ significantly (*p* = 0.289). The CRP comparison included 197 patients; all other between-group comparisons included the full cohort of 206 patients (Table 1).

#### 3.1.2. Factors Associated with Re-Amputation Group Membership: Binary Logistic Regression

In the primary model, age (adjusted OR = 1.034, 95% CI: 1.010–1.058; *p* = 0.006) and CRP (adjusted OR = 1.006, 95% CI: 1.003–1.010; *p* = 0.001) were associated with re-amputation group membership, whereas NLR was not (*p* = 0.834). Replacing NLR with MLR or PLR did not materially alter the associations of age and CRP; neither MLR (*p* = 0.176) nor PLR (*p* = 0.446) was associated with group membership after adjustment. The Hosmer–Lemeshow tests showed no evidence of lack of fit in the three models (Table 2). In both missing-CRP scenarios, age and CRP remained associated with re-amputation group membership, whereas NLR did not.

#### 3.1.3. Linearity in the Logit

None of the Box–Tidwell interaction terms were statistically significant at the Bonferroni-adjusted threshold of *p* = 0.0167 (observed *p* = 0.164–0.466). Therefore, no evidence of departure from the linearity-in-the-logit assumption was identified in any of the three models (Table 3).

#### 3.1.4. Multicollinearity Diagnostics

Tolerance values ranged from 0.837 to 0.998, and VIF values ranged from 1.002 to 1.194. These findings indicated no evidence of problematic multicollinearity in any of the three models (Table 4).

#### 3.1.5. ROC Analysis and Cut-Off Values

**Figure 1:**
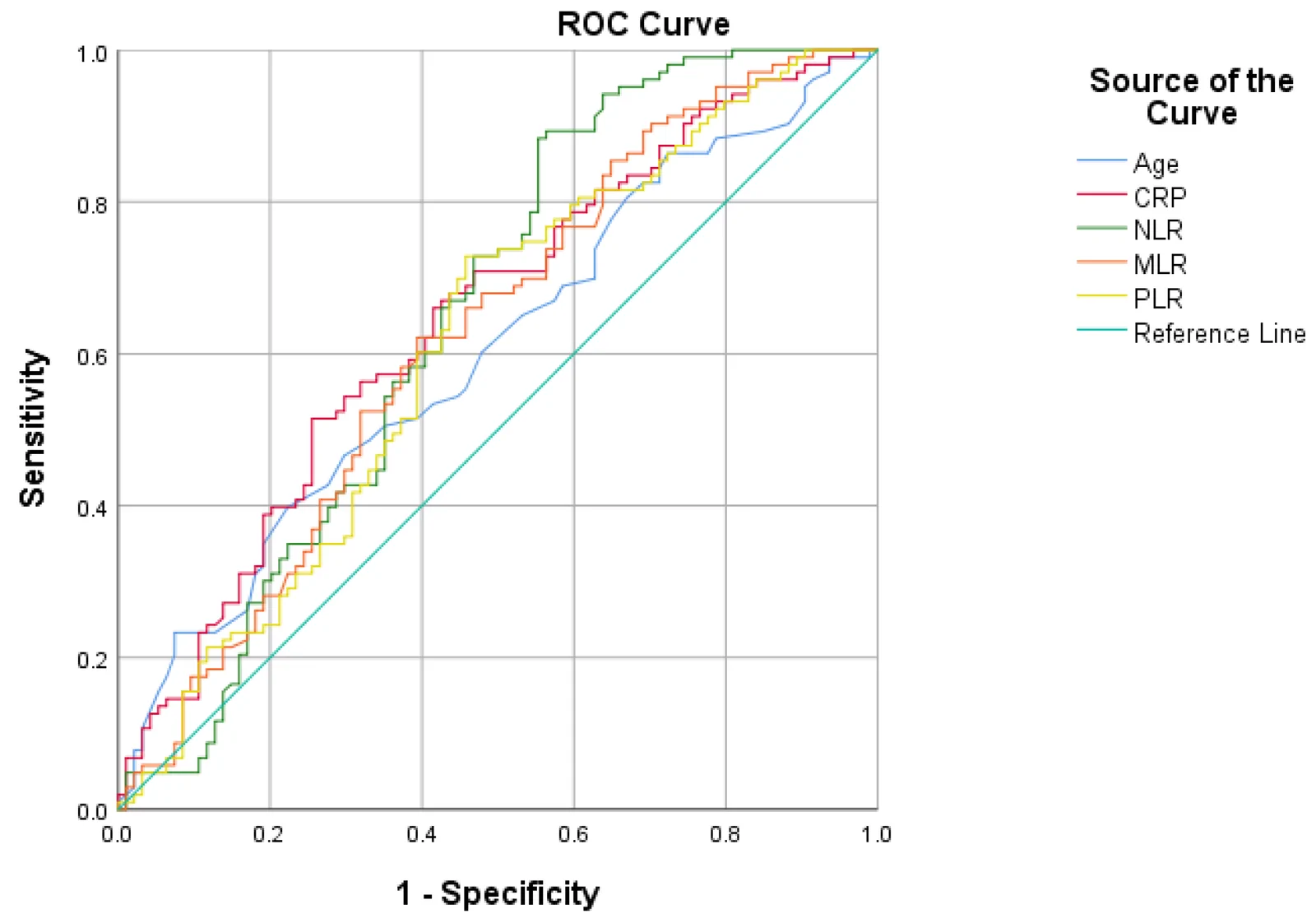
Receiver operating characteristic curves for age, CRP, NLR, MLR, and PLR in discriminating membership in the re-amputation group. All curves were generated using the same complete-case sample of 197 patients.

ROC curve analysis was performed in the common complete-case sample of 197 patients to ensure that all variables were evaluated in the same individuals. CRP and NLR showed the highest discriminatory performance, although both AUC values were modest (AUC = 0.647). At the optimal cutoffs, NLR ≥ 4.40 provided higher sensitivity (88.3%) but lower specificity (44.7%), whereas CRP ≥ 152.2 mg/L provided 51.5% sensitivity and 74.5% specificity. The AUC values for age, MLR, and PLR ranged from 0.604 to 0.624, indicating limited discrimination overall (Figure 1 and Table 5).

## 4. Discussion

In this study, patients with documented re-amputation were older and had higher preoperative CRP, WBC, neutrophil, monocyte, NLR, MLR, and PLR values, whereas lymphocyte counts were lower. In the exploratory models, age and CRP remained associated with re-amputation group membership after adjustment for one inflammatory ratio at a time. NLR, MLR, and PLR did not show statistically significant adjusted associations in their respective models. These findings suggest that differences observed in individual blood-cell ratios should be interpreted alongside age and CRP rather than in isolation.

In patients with diabetic foot disease, inflammatory markers play a role in the re-amputation process alongside clinical findings. Therefore, hematological and biochemical markers have received increasing attention in recent years due to their potential to indicate systemic inflammation. Important markers such as CRP, WBC, LYM, NEU, MON, PLT, NLR, and MLR are valuable indicators because of their accessibility and cost-effectiveness. NLR has been studied in patients with chronic inflammatory diseases, and diabetic foot patients [6,7,8,12,13]. Furthermore, existing literature indicates that NLR and PLR values are significant predictors of mortality in patients who have undergone amputation due to diabetic foot ulcers [14]. Alongside these markers, the Delta Neutrophil Index (DNI)—another hematological parameter—has also been found to be a significant prognostic indicator for early-stage revision processes in patients undergoing non-traumatic below-knee amputations [15]. In this investigation, we compared metrics such as age, WBC, CRP, LYM, NEU, MON, PLT, NLR, and MLR, along with their interrelations, and examined how these metrics influence the re-amputation process.

Many factors contribute to amputation in diabetic foot patients. These include male gender, smoking, history of amputation, presence of osteomyelitis, and peripheral arterial disease [16]. However, their exact effect on the algorithm is not known. One of the most important reasons for re-amputation is previous amputation. There are publications reporting that the rate of re-development of an ulcer in amputated patients within 1 year is between 20–58% [17].

Risk factors for re-amputation have been extensively studied in the literature. In the study by Gülcü et al., it was suggested that HbA1c, CRP, and creatinine levels may be associated with re-amputation; in particular, it was reported that low albumin and high HbA1c levels play a role in this process [9]. On the other hand, Dai et al. demonstrated that glycated hemoglobin A1c (HbA1c), ankle-brachial index (ABI), C-reactive protein (CRP), and smoking status influence the risk of re-amputation, and noted that examining additional parameters could provide new insights in the future [18]. In addition, another study has suggested that an elevated serum CRP-to-albumin ratio could serve as a potential predictor of amputation [19]. Recent literature also highlights that while CRP and NLR levels remain elevated in diabetic patients during both the pre- and post-amputation periods, a significant decline in these values is observed following the amputation procedure [20].

In recent literature, neutrophil count and white blood cell (WBC) values have been identified as significant predictors of postoperative complications following diabetic foot amputation. Considering re-amputation as a postoperative complication, the findings in our study regarding neutrophil and WBC values are highly consistent with current literature [21].

Patients with documented re-amputation had higher preoperative CRP levels, as well as higher values for several blood-cell counts and derived inflammatory ratios, than those without documented re-amputation. In the exploratory multivariable models, age and CRP were independently associated with re-amputation group membership. In contrast, NLR, MLR, and PLR were not significantly associated with re-amputation after adjustment for age and CRP when evaluated separately in the respective models. Absolute blood-cell counts were not included in these adjusted analyses.

Chronic low-grade inflammation associated with diabetes and peripheral neuropathy may contribute to the inflammatory background in diabetic foot disease. Superimposed infection or tissue injury near the index amputation could generate an additional acute-phase response. IL-6 stimulates hepatic CRP production, while TNF-α participates in inflammatory pathways associated with diabetic foot tissue damage [22,23].

CRP may therefore reflect aspects of the systemic inflammatory burden that are not fully captured by ratios derived from circulating blood-cell counts. This may partly explain its association with re-amputation in the adjusted models; however, this finding should not be interpreted as evidence of an established biological mechanism.

This interpretation should be approached with caution. We did not measure IL-6, TNF-α, the extent of tissue necrosis, or infection severity and therefore could not determine whether acute inflammation was superimposed on chronic inflammatory processes in individual patients. Furthermore, CRP and NLR showed identical individual-marker AUC values (0.647), indicating that our findings do not demonstrate superior discriminatory performance of CRP. The observed association may also reflect greater baseline disease severity or other unmeasured clinical factors. Although Vatankhah et al. [24] reported NLR as an independent predictor of wound healing in patients with diabetic foot ulcers, NLR was not significantly associated with documented re-amputation after adjustment for age and CRP in our cohort. The different clinical endpoints may contribute to this discrepancy. In the multivariate logistic regression analysis, it was found that age and CRP remained associated with re-amputation group membership after adjustment for the variables included in the models, whereas NLR, MLR, and PLR did not show statistically significant adjusted associations in their respective models. According to the results of our study, each one-unit increase in age was associated with a 3.4% increase in the risk of re-amputation, while each one-unit increase in CRP was associated with a 0.6% increase in risk. Serum CRP levels are acknowledged as one of the essential indicators of inflammatory activity. However, there is no comprehensive consensus in the literature regarding the prognostic value of this parameter. While Volaco et al. noted that CRP levels might be significant in forecasting progression to amputation, several studies have reported that the sensitivity of WBC and CRP is restricted in this regard [25,26]. In another study, it was found that CRP and CRP/albumin levels were significant factors influencing mortality in patients who had undergone amputation and re-amputation [27]. In our study, both WBC and CRP differed between the groups. CRP remained associated with re-amputation group membership in the adjusted models; however, WBC was not included in these models. Therefore, our findings do not establish that CRP is a stronger or more stable marker than WBC. Although NLR, MLR, and PLR differed between the groups, none showed a statistically significant association after adjustment for age and CRP in their respective models. All five measures examined in the ROC analysis—age, CRP, NLR, MLR, and PLR—showed limited discrimination when evaluated individually. CRP and NLR had identical AUC values; thus, these results do not support a claim of superior discriminatory performance for CRP.

## 5. Limitations

This study has several limitations. Its retrospective, single-center design and the selection of equal numbers of patients from the two outcome groups limit generalizability and prevent estimation of the population frequency of re-amputation. The adjusted models did not include several clinically relevant factors, including infection severity, osteomyelitis, glycemic control, renal function, nutritional status, smoking, perfusion status, and prior revascularization. Although amputations performed primarily for isolated arterial occlusion were excluded, perfusion was not assessed systematically across the cohort, and concomitant peripheral arterial disease could not be adequately characterized. Residual confounding may therefore explain some of the observed associations. The anatomical levels of the index and subsequent amputations were not recorded in the analytic dataset, preventing analysis of minor-to-major progression or differences by initial amputation level.

Re-amputation status was determined from available hospital records without a uniform minimum follow-up period. Event and last-contact dates were not consistently available, so some patients classified as having no documented re-amputation may have undergone the procedure later or elsewhere. Time-dependent re-amputation risk could not be estimated. Changes in diabetic foot care during the 2013–2024 inclusion period were not accounted for. Although blood samples were collected within 24 h before the index amputation, sampling was not standardized relative to antibiotic or anti-inflammatory treatment, and pretreatment exposure was not systematically recorded. Finally, CRP was missing for nine patients, all in the group without documented re-amputation. Our sensitivity analyses explored the effect of these missing values but could not eliminate potential bias. The modest sample size and limited, unvalidated discrimination of the individual markers further restrict their clinical application. Finally, we examined a limited panel of routinely available inflammatory markers; other biomarkers and measures of disease severity were not assessed.

## 6. Conclusions

In summary, NLR, MLR, and PLR differed between patients with and without documented re-amputation, but showed limited discrimination when assessed individually and were not significantly associated with group membership after adjustment for age and CRP. Age and CRP remained associated with documented re-amputation in the exploratory models. Larger studies with consistent follow-up and relevant clinical variables are needed to determine whether these findings can inform risk assessment.

## Figures and Tables

**Table 1:** Comparison of demographic, hematological, and inflammatory parameters between patients with and without re-amputation.

	Group						
	No-Re Amputation (0)			Re-Amputation (1)			
	*n*	Mean ± SD	Median (25–75%)	*n*	Mean ± SD	Median (25–75%)	*p*
Age	103	65.18 ± 12.91	66.00 (54.00–75.00)	103	70.08 ± 13.07	72.00 (60.00–80.00)	0.009 *
WBC	103	13.92 ± 6.71	12.05 (9.50–15.70)	103	16.04 ± 6.91	14.69 (10.55–19.30)	0.006 *
LYM	103	1.73 ± 0.76	1.61 (1.20–2.12)	103	1.52 ± 0.66	1.46 (1.06–1.84)	0.031 *
NEU	103	11.01 ± 6.70	9.02 (6.40–13.65)	103	13.30 ± 6.66	11.49 (8.40–16.70)	0.002 *
MON	103	1.04 ± 0.90	0.92 (0.64–1.20)	103	1.10 ± 0.46	1.06 (0.77–1.36)	0.040 *
PLT	103	378.28 ± 146.32	380.00 (262.00–500.00)	103	406.50 ± 154.50	391.00 (307.00–467.00)	0.289
CRP	94	113.58 ± 85.44	101.65 (38.58–158.99)	103	157.56 ± 89.96	153.00 (86.25–221.00)	<0.001 *
NLR	103	8.73 ± 10.27	5.51 (3.29–10.35)	103	10.54 ± 8.52	7.80 (5.22–14.30)	<0.001 *
MLR	103	0.68 ± 0.44	0.57 (0.39–0.88)	103	0.82 ± 0.44	0.74 (0.51–0.95)	0.004 *
PLR	103	264.87 ± 180.60	208.51 (145.23–339.65)	103	305.30 ± 171.33	265.67 (196.45–381.20)	0.005 *

Data are presented as mean ± standard deviation and median (25th–75th percentile). Between-group comparisons were performed using the Mann–Whitney U test. * *p* < 0.05.

**Table 2:** Binary logistic regression models for re-amputation group membership.

Model	Variable	B	SE	OR (Exp(B))	95% CI	*p*
Model 1	Age	0.033	0.012	1.034	1.010–1.058	0.006 *
	CRP	0.006	0.002	1.006	1.003–1.010	0.001 *
	NLR	−0.004	0.017	0.996	0.963–1.031	0.834
Model 2	Age	0.034	0.012	1.035	1.010–1.060	0.005 *
	CRP	0.006	0.002	1.006	1.002–1.009	0.002 *
	MLR	0.486	0.360	1.626	0.803–3.291	0.176
Model 3	Age	0.033	0.012	1.034	1.010–1.058	0.006 *
	CRP	0.006	0.002	1.006	1.002–1.010	0.001 *
	PLR	0.001	0.001	1.001	0.999–1.002	0.446

Model 1: age, CRP, and NLR; Model 2: age, CRP, and MLR; Model 3: age, CRP, and PLR. All models were fitted using the Enter method and included 197 complete cases. Re-amputation was coded as 1 and no re-amputation as 0. The Hosmer–Lemeshow tests did not indicate evidence of lack of fit for Model 1 (χ^2^ = 10.965, df = 8, *p* = 0.204), Model 2 (χ^2^ = 6.216, df = 8, *p* = 0.623), or Model 3 (χ^2^ = 4.355, df = 8, *p* = 0.824). OR: odds ratio; CI: confidence interval; SE: standard error. * *p* < 0.05.

**Table 3:** Assessment of linearity in the logit using Box–Tidwell interaction terms.

Model	Interaction Term	*p*	Bonferroni Threshold
Model 1	Age × ln(Age)	0.369	0.0167
	CRP × ln(CRP)	0.315	0.0167
	NLR × ln(NLR)	0.172	0.0167
Model 2	Age × ln(Age)	0.370	0.0167
	CRP × ln(CRP)	0.329	0.0167
	MLR × ln(MLR)	0.466	0.0167
Model 3	Age × ln(Age)	0.390	0.0167
	CRP × ln(CRP)	0.355	0.0167
	PLR × ln(PLR)	0.164	0.0167

Model 1: age, CRP, and NLR; Model 2: age, CRP, and MLR; Model 3: age, CRP, and PLR. Linearity in the logit was evaluated by including an interaction term between each continuous predictor and its natural logarithm. A Bonferroni-adjusted significance threshold of *p* = 0.0167 (0.05/3) was applied within each model. None of the interaction terms were statistically significant, indicating no evidence of violation of the linearity-in-the-logit assumption. All analyses included the same 197 complete cases. CRP: C-reactive protein; NLR: neutrophil-to-lymphocyte ratio; MLR: monocyte-to-lymphocyte ratio; PLR: platelet-to-lymphocyte ratio.

**Table 4:** Multicollinearity diagnostics for the binary logistic regression models.

Model	Predictor	Tolerance	VIF
Model 1	Age	0.997	1.003
	CRP	0.837	1.194
	NLR	0.838	1.193
Model 2	Age	0.998	1.002
	CRP	0.896	1.116
	MLR	0.897	1.115
Model 3	Age	0.998	1.002
	CRP	0.950	1.052
	PLR	0.952	1.051

Model 1: age, CRP, and NLR; Model 2: age, CRP, and MLR; Model 3: age, CRP, and PLR. Multicollinearity diagnostics were calculated using the same complete-case sample for all models (*n* = 197). Tolerance values > 0.20 and VIF values < 5 were considered indicative of the absence of problematic multicollinearity. CRP: C-reactive protein; NLR: neutrophil-to-lymphocyte ratio; MLR: monocyte-to-lymphocyte ratio; PLR: platelet-to-lymphocyte ratio; VIF: variance inflation factor.

**Table 5:** ROC curve analysis of age and inflammatory parameters for discrimination of re-amputation group membership.

Variable	AUC	SE	95% CI	*p*	Optimal Cutoff	Sensitivity	Specificity	Youden Index
Age	0.604	0.040	0.525–0.682	0.012	≥75.5 years	39.8%	77.7%	0.175
CRP	0.647	0.039	0.570–0.724	<0.001	≥152.2 mg/L	51.5%	74.5%	0.259
NLR	0.647	0.040	0.568–0.726	<0.001	≥4.40	88.3%	44.7%	0.330
MLR	0.624	0.040	0.545–0.702	0.003	≥0.628	62.1%	60.6%	0.228
PLR	0.614	0.040	0.535–0.693	0.006	≥211.00	72.8%	54.3%	0.271

## Data Availability

The data presented in this study are available on reasonable request from the corresponding author. The data are not publicly available due to privacy and ethical restrictions related to patient confidentiality and institutional review board regulations.

## Abbreviations

The following abbreviations are used in this manuscript:

ABI	Ankle-Brachial Index
AUC	Area Under the Curve
CI	Confidence Interval
CRP	C-Reactive Protein
DNI	Delta Neutrophil Index
HIMS	Hospital Information Management System
LYM	Lymphocyte
MLR	Monocyte-to-Lymphocyte Ratio
MON	Monocyte
NEU	Neutrophil
NLR	Neutrophil-to-Lymphocyte Ratio
OR	Odds Ratio
PLR	Platelet-to-Lymphocyte Ratio
PLT	Platelet
ROC	Receiver Operating Characteristic
SD	Standard Deviation
SPSS	Statistical Package for the Social Sciences
WBC	White Blood Cell
